# Pilose antler extracts promotes hair growth in androgenetic alopecia mice by activating hair follicle stem cells via the AKT and Wnt pathways

**DOI:** 10.3389/fphar.2024.1410810

**Published:** 2024-07-09

**Authors:** Fenglong Wang, Gaiying He, Menghua Liu, Yanan Sun, Shuhua Ma, Zhenxiao Sun, Yi Wang

**Affiliations:** ^1^ School of Life Sciences, Beijing University of Chinese Medicine, Beijing, China; ^2^ Experimental Research Center, China Academy of Chinese Medical Sciences, Beijing, China

**Keywords:** androgenetic alopecia, hair growth, pilose antler extracts, hair follicle stem cell, AKT/Wnt pathway

## Abstract

**Background:** Angrogenetic alopecia (AGA) is one of the most prevalent hair loss disorders worldwide. The hair follicle stem cell (HFSC) is closely related to the formation of hair follicle (HF) structure and HF self-renewal. The activation of HFSC in AGA is critical for hair growth. Pilose antler has been reported to have hair growth-promoting activity, but the mechanism of action on AGA and HFSC has not been reported.

**Methods:** We previously extracted an active component from the pilose antler known as PAEs. In this study, we conducted experiments using AGA mice and HFSC. The effects of PAEs on hair growth in AGA mice were firstly detected, and then the mechanisms of PAEs for AGA were predicted by integrating network pharmacology and *de novo* transcriptomics data of pilose antler. Finally, biological experiments were used to validate the molecular mechanism of PAEs in treating AGA both *in vivo* and *in vitro*.

**Results:** It was found that PAEs promoted hair regrowth by accelerating the activation of anagen, delaying the anagen-catagen transition. It also alleviated the morphological changes, such as hair shortening, thinning, miniaturization, and HF number reduction, and regulated the hair regeneration process of four subtypes of hair. We further found that PAEs could promote the proliferation of HFSC, outer root sheath (ORS) cells, and hair bulb cells in AGA mice. We then integrated network pharmacology and pilose antler transcriptomics data to predict that the mechanism of PAEs treatment in AGA mice is closely related to the PI3K-AKT/Wnt-β-Catenin pathways. Subsequently, it was also verified that PAEs could activate both pathways in the skin of AGA mice. In addition, we found that PAEs perhaps increased the number of blood vessels around dermal papilla (DP) in experiments *in vivo*. Meanwhile, the PAEs stimulated the HFSC proliferation *in vitro* and activated the AKT and Wnt pathways. However, the proliferative activity of HFSC was inhibited after blocking the Wnt pathway and AKT activity.

**Conclusion:** This study suggests that the hair growth-promoting effect of PAEs in AGA mice may be closely related to the stimulation of the AKT and Wnt pathways, which in turn activates the proliferation of HFSC.

## 1 Introduction

Androgenetic alopecia (AGA) is a progressive hair reduction disease, showing an increasing tendency in younger ages in recent years. The primary cause is the disturbance of the hair cycle, resulting in gradual hair follicle (HF) shrinkage (Morgan and Rose, 2003; Lolli et al., 2017). Finasteride and minoxidil (MIN) are approved by the FDA for AGA treatment (Kelly et al., 2016; Lee et al., 2018). However, finasteride-induced adverse effects are observed in clinics, including gynecomastia and sexual dysfunction (Mysore and Shashikumar, 2016; Dhurat et al., 2020). MIN may lead to local irritation, itching, dryness, and erythema (Diani et al., 1992; Rogers and Avram, 2008). Thus, developing effective therapeutic strategies for AGA therapies is urgently needed.

Traditional Chinese Medicine (TCM) is receiving increasing attention for potential therapeutic developments in treating AGA (Zhang et al., 2016). The pilose antler, recognized as the osseous cranial appendage of male sika deer (*Cervus nippon* Temminck) or red deer (*Cervus elaphus* Linnaeus), has been used as medicine in China for a long history. It is unique among mammalian accessory organs due to its remarkable ability to regenerate completely every year (Wu et al., 2013), which possesses properties enhancing overall vitality and energy (Wu et al., 2013; Huang et al., 2014; Chinese National Pharmacopoeia Committee, 2020). Pilose antler contains biologically active components including proteins, peptides, amino acids, and a variety of growth factors (such as insulin-like growth factor (IGF), vascular endothelial growth factor (VEGF), fibroblast growth factor (FGF), and nerve growth factor (HGF), which have physiological activities such as promoting wound healing, enhancing immunity, and promoting cell regeneration (Sui et al., 2014). Furthermore, in recent studies, some certain bioactive protein extracts from pilose antler were proven to be delivered through the skin, efficiently fostering hair growth (Li et al., 2014; Tansathien et al., 2021), and it has been shown that uridine, a metabolite in pilose antler, has a significant role in hair growth (Liu et al., 2022). However, until now, it’s unclear whether pilose antler could alleviate the AGA disease.

Hair follicle stem cells (HFSC) are located in the bulge formed by the outer root sheath (ORS) and play an important role in the processes involved in the regeneration of the HF and the regulation of the hair cycle (Alonso and Fuchs, 2006). Besides, the interactions between HFSC and hair bulb cells and ORS cells are crucial for the formation of HF structure (Fuchs, 2007). Androgens inhibit the ability of HFSC to activate and differentiate by affecting the microenvironment around the HF, resulting in a failure of normal hair growth and renewal (Leiros et al., 2017). Recent studies have found that stimulation of the PI3K-AKT/Wnt-β-Catenin Pathways is strongly associated with HFSC activation and HF formation (Qiu et al., 2017; Wang et al., 2017; Jin et al., 2021). In addition, the pilose antler has a regulatory effect on the activation of the PI3K-AKT/Wnt-β-Catenin Pathways (Mount et al., 2006; Lee and Choi, 2024; Orassay et al., 2024), however, whether it regulates HFSC activation through this pathway has not been reported. Besides, in our previous study, active components extracted from pilose antler were known as PAEs. We found that PAEs promoted the growth of blood vessels and collagen fibers during the skin healing process in rats (Li et al., 2023), and alleviated the adverse effects of photoaged skin exposed to ultraviolet radiation (Tang et al., 2022). Based on the above studies, we speculate that PAEs has a close link to the activation, proliferation, and differentiation of stem cells in the skin, and whether it can promote the hair growth in AGA through the activation of HFSC has not been explored.

Herein, this work is to investigate the hair regrowth effect of PAEs against AGA and identify the underlying mechanisms. We utilized TES-induced AGA mice and HFSC as *in vivo* and *in vitro* experimental study subjects. Firstly, we intervened AGA mice with PAEs to observe whether it has hair pro-growth effect. Secondly, the effects of PAEs on hair morphology, hair cycle, the proliferation of HFSC, and blood vessels around dermal papilla (DP) in AGA mice were examined. Then we integrated network pharmacology and transcriptome analysis of pilose antler to predict the mechanism of PAEs in the treatment of AGA, for which we also performed a validation *in vivo*. Finally, we also investigated the mechanism of PAEs in promoting the proliferation of HFSC *in vitro* cell assays.

## 2 Materials and methods

### 2.1 PAEs’ preparation

PAEs were prepared by superfine crushing of pilose antler and then extracted with acetic acid. Brifly, fresh pilose antlers were ultra-finely grinded, crushed, and then washed till no blood remained. After that, it was submerged in water and the pH was adjusted to 3.5 using glacial acetic acid for an entire night. Then collect the supernatant and put in a dialysis bag and desalted to reach a neutral pH, and the liquid was concentrated with a rotary evaporator. The precipitates were removed by centrifugation, and the supernatant was then collected and preserved using a lyophilizer for freeze-drying. We then diluted the PAEs lyophilized powder to different drug concentrations using a gel prepared with CMC-Na, glycerol, and pure water.

### 2.2 Analysis of *De novo* transcriptome data from pilose antler

The pilose antler tissue samples were ground and crushed by adding liquid nitrogen, and then the lysis solution was added to make the cells fully lysed, and the RNA was extracted and purified. The quality of total RNA was checked using an Agilent 2100 Bioanalyzer. Then the mRNA with polyA tail was enriched by magnetic beads with OligodT, and the obtained RNA was fragmented by interrupting the buffer, and then the cDNA was synthesized by reverse transcription with the addition of random N6 primers and modified. Then PCR amplification was carried out by the added specific primers, and after the PCR products were heat denatured into single stranded, they were looped with a piece of bridge primer to obtain a single-stranded circular DNA library, and finally up-sequencing was carried out.

Reads with low quality, splice contamination, and excessive unknown base N content are then filtered out of the raw data from sequencing, and the filtered data are called clean reads. The clean reads were *de novo* assembled using Trinity, and then clustered and de-redundantly assembled transcripts were clustered and de-redundantly assembled using Tgicl to obtain the Unigene. Bowtie2 (http://bowtie-bio.sourceforge.net/) was applied to align the clean reads to the Unigene, and RSEM (http://deweylab.biostat.wisc.edu/) was used to calculate the gene expression levels in the pilose antler samples. Finally, we integrated the network pharmacology approach and *de novo* transcriptomics data of pilose antler to speculate on the potential mechanism of PAEs for AGA treatment.

### 2.3 The active ingredients of PAEs and AGA disease target prediction

Using Androgenetic alopecia as the keyword, we collected AGA disease-related gene information in DisGeNET database (https://www.disgenet.org/) and GeneCards database (https://www.genecards.org/). Then the jvenn (http://www.bioinformatics.com.cn/static/others/jvenn/example.html) was applied to take the intersection analysis between pilose antler transcriptomics data and AGA disease targets to obtain the key targets of PAEs for AGA treatment.

### 2.4 Functional enrichment analysis of core network targets

Gene Ontology (GO) and Kyoto Encyclopedia of Genes and Genomes (KEGG) pathway enrichment analyses of key targets of PAEs for AGA treatment were performed using the Bioinformatics online tool (https://bioinformatics.com.cn). The GO enrichment analyses included biological processes (BP), cellular component (CC), and molecular function (MF), while the KEGG enrichment mainly showed pathway-related content.

### 2.5 Experimental animals

The 6-week-age C57BL/6 male mice were housed in the environment of a 12-h cycle of light and dark, with an ambient temperature of 20°C–24°C and 45%–65% humidity. Electric hair scissor (Codos, Shenzhen, China) and commercial hair removal gel (veet, Reckitt Benckiser) were used to gently depilate the dorsal hair.

Seven groups of six-week-old mice were randomly assigned: Control group (CON), TES-induced AGA model group (TES-group), PAEs gel group (Concentration:1, 2, 4, 8 mg/mL), and positive control group (MIN).

Existing studies have shown that topical application of TES can mimic the pathological characteristics of AGA (Zhang et al., 2016; Lin et al., 2019; Yuan et al., 2021; Ding et al., 2024). Compared to intraperitoneal and subcutaneous injection methods, topical application of TES reduces stress and pain in mice while creating a localized high concentration of hormone environment on the dorsal skin. The AGA mouse model was established and presented with a few minor adjustments from Zhang in this work (Zhang et al., 2016). In brief, the TES (Nature standard, ST053801, Shanghai, China) solution (1 mg/mL) was prepared in 75% ethanol solution. From the second day, mice in each group were applied topically to the back with 150 μL TES solution for 26 straight days except the CON group. Mice in the CON group were received 75% ethanol solution. Subsequently, 100 μL of PAEs and MIN were applied topically after 2 h of TES treatment. The PAEs group received a mixture of PAEs gel at a concentration of 1, 2, 4, and 8 mg/mL, while the MIN group received 5% MIN.

### 2.6 The hair growth effects of PAEs in AGA mice

We observed the darkness of the mice’s skin color daily which indicated the hair cycle conversion. The occurrences of skin darkening and hair growth were recorded on days 1, 4, 6, 8, 12, 18, 22, and 26 post-topical application. Digital photographs were taken at these time points, and the level of hair growth and pigmentation was quantified using ImageJ software (Son et al., 2018) in a specific region of the back skin (3.0 × 4.0 cm). Meanwhile, the weight of the mice was recorded at each time point.

### 2.7 Medical electronic dermoscopy

Micro-characteristic changes in AGA mice were observed in a representative area of dorsal skin under ×60 objective using medical electronic dermoscopy (MED) (BN-WG-1001, Nanjing). Furthermore, the morphology of hair subtype and hair shaft length were recorded through 180 × and 160 × objective respectively.

### 2.8 Western blotting

Mice’s dorsal skin and HFSCs were collected for immunoblotting analysis, and proteins were separated using a polyacrylamide gel (GenScript, M00654), and then they were moved onto a polyvinylidene fluoride membrane (Millipore, ISEQ00010). The appropriate primary monoclonal antibody was incubated on the membrane for a whole night at 4 °C after blocking with blocking solution (NCM Biotech, P30500), then exposed to a secondary antibody that had been coupled to horseradish peroxidase. Using enhanced chemiluminescence (Millipore, WBLUR0500) substrates and an imaging system (BIO-RAD, ChemiDoc), protein bands were identified, and quantified by ImageJ software. Every example blot’s optical density was calibrated using internal control proteins (Gapdh, Proteintech, 10494, 1:5000). The antibodies dilutions were as followings: AKT (rabbit, Abclonal, AP1208, 1:1000), p-AKT-Ser473 (rabbit, Abclonal, AP0637 1:1000), p-PI3K (rabbit, Abcam, ab182651, 1:2000), PI3K (rabbit, Abcam, ab227204 1:2000), p-GSK3β-Ser9 (rabbit, Abclonal, AP1088, 1:2000), GSK3β (rabbit, Abclonal, A11731, 1:2000), β-catenin (rabbit, Abclonal, A19657, 1:2000), WNT3A (rabbit, Abclonal, A0642, 1:2000), WNT10B (rabbit, Abclonal, A16717, 1:2000).

### 2.9 RT-qPCR

Total RNA was obtained using the extract kit. Subsequently, the Master Mix (TOYOBO, FSQ-201) was used to make the mRNA become complementary DNA (cDNA), and the resultant cDNA was used as the template. The 40 cycles in the thermal cycling program were set following the instructions. Using the 2^−ΔΔCt^ technique, relative mRNA expression levels in each sample were measured by normalizing to Gapdh. The sequences of primer are displayed in Table 1.

**TABLE 1:** List of sequences of forward and reverse primers.

Primer name		Sequence
*Axin1*	F	CCA​GCC​ACC​AGT​GCC​AAT​GAC
	R	TCC​ATC​CAC​ACT​ACT​GTC​CGT​AAG​G
*Axin2*	F	GAG​CGG​CAG​AGC​AAG​TCC​AAG
	R	CGC​ACA​GGC​AGA​CTC​CAA​TGG
*TCF-7*	F	GGA​AAG​AAG​AAG​AGG​CGG​TCA​AGG
	R	GAG​CAC​TGT​CAT​CGG​AAG​GAA​CG
*c-Jun*	F	CTT​CTA​CGA​CGA​TGC​CCT​CAA​CG
	R	GCC​AGG​TTC​AAG​GTC​ATG​CTC​TG
*Lef-1*	F	CAC​ACA​ACT​GGC​ATC​CCT​CAT​CC
	R	TGG​GCT​CCT​GCT​CCT​TTC​TCT​G
*Survivin*	F	TCA​TCC​ACT​GCC​CTA​CCG​AGA​AC
	R	TCC​CAG​CCT​TCC​AAT​TCC​TTA​AAG​C
*Lgr6*	F	CAC​CTC​TGG​CTG​GAT​GAC​AAT​GC
*Cyclind-1*	R	TAG​TCA​GGG​ATG​TGG​CGG​ATA​TGG
	F	GGA​AAC​GCC​GAC​AGT​GTC​TTC​TC
	R	GTT​GTG​TTG​CTG​CTG​TGC​TTG​G

### 2.10 Hematoxylin and eosin (H&E) staining

Mice’s dorsal skin was harvested on days 1, 4, 6, 8, 12, 18, 22, and 26. The samples were preserved in 4% paraformaldehyde for a full day, then dehydrated and paraffin-embedded to produce 4 μm slices. Following deparaffinization using xylene and rehydration through varying ethanol concentrations, then stained using the kit (Solarbio, G1120). Microscopic observation and image acquisition were performed utilizing a microscope (Olympus, CKX41). The Image-Pro Plus software was used to measure the skin and fat layer’s thickness, HFs’ length, and hair bulb’s diameter.

### 2.11 Immunofluorescence staining

For immunofluorescence analysis, back skin tissues from mice at day 12 were embedded in OCT compound (Tissue Tek, 4583, American), and 10 μm frozen sections were made using a freezing microtome (CryoStar NX70, Epredia, China). These sections were permeabilized for half an hour, then treated for 1 h with immunofluorescence blocking solution (5% BSA in TBS), and with primary antibodies and secondary fluorescence-conjugated antibodies: anti-chicken (Bioss, bs0310G, 1:500), anti-rabbit (Abcam, ab150077, 1:500) at RT before DAPI staining (Yuanye, Shanghai, S19119). Images were captured by confocal microscope (Olympus, FV1000MPE). The dilutions of the antibodies were as follows: KI67 (rabbit, Abcam, ab15580, 1:500), Cytokeratin 15 (K15) (rabbit, Abcam, ab52816, 1:200), Cytokeratin 14 (K14) (chicken, Biolgend, 906004, 1:100), β-Catenin (mouse, Abclonal, A19657, 1:200), p-AKT-Thr308 (rabbit, CST, 13038S), CD31 (R&D Systems, AF3628). We conducted a statistical analysis of CD31-labeled blood vessels around DP following Deng et al. (2022).

### 2.12 HFSC culture

Rat primary immortalized HFSCs were purchased from Guangzhou Chuyuanyun Medical Technology Co. The cell culture medium was consisted of 2% FBS, 1% penicillin-streptomycin-amphotericin B solution, and 1% culture additives, and the cells were cultured in a humid environment at 37°C with 5% CO_2_.

### 2.13 Cell proliferation assay

The effect of PAEs on HFSC proliferation at different drug concentrations was tested by Cell Counting Kit 8 (CCK8; Biosharp, China). Cells were inoculated in 96-well plates at a density of 5 × 10^3^ cells/well. The next day, the culture was continued for 24h after adding PAEs or PAEs plus inhibitors (IWP-2 and API-2 block the Wnt pathway and AKT activity, respectively), and then the CCK8 solution was added to detect the absorbance.

### 2.14 Cell clone formation assay

Cell clone formation assay was used to evaluate the cell proliferation and clone-forming ability of HFSC after PAEs treatment with or without AKT or Wnt pathway inhibitors. Cells were inoculated in 6-well plates at a density of 500 cells/well, and inhibitors (IWP-2 and API-2) and PAEs were treated alone or jointly the next day and cultured continuously for 14 days. Then the cells were fixed with 4% paraformaldehyde and then stained with crystal violet solution. We observed the cells under a microscope and took photographs, defining cluster of more than 50 cells as a cell colony. We ensure that each counted cluster contains over 50 cells and consistently apply this method across all colonies to maintain uniformity and accuracy in the experiment. Using ImageJ software, we counted the colonies and calculated the colony formation rate.

### 2.15 Statistical analysis

Graph Prism 9 was used to determine the statistical differences in One-way ANOVA analysis. The data were expressed as mean ± standard deviation (SD). The caption for every figure provides information on the number of animals and *p* values. *p* < 0.05 was considered significant.

## 3 Results

### 3.1 PAEs activated the entry of anagen and delayed catagen to enhance hair growth in AGA mice

Beginning in the seventh week of postnatal life, the mice enter the second telogen phase of hair growth on the back skin, which lasts around 6 weeks (Müller-Röver et al., 2001). To confirm PAEs have an impact on hair development, we carried out a pre-experiment. PAEs gel was applied at concentrations of 1, 2, 4, and 8 mg/mL to the back of AGA mice at the seventh week of postnatal life. We found PAEs promoted hair growth in AGA mice, with the greatest benefit occurring at the concentration of 8 mg/mL, which was equivalent to the effect of the positive control MIN group (Supplementary Figures S1A, B). These observations indicated that PAEs have a hair growth-promoting effect on AGA mice. 8 mg/mL PAEs was used in subsequent experiments to reveal the underlying mechanism of hair regrowth ability in AGA mice.

**FIGURE 1:**
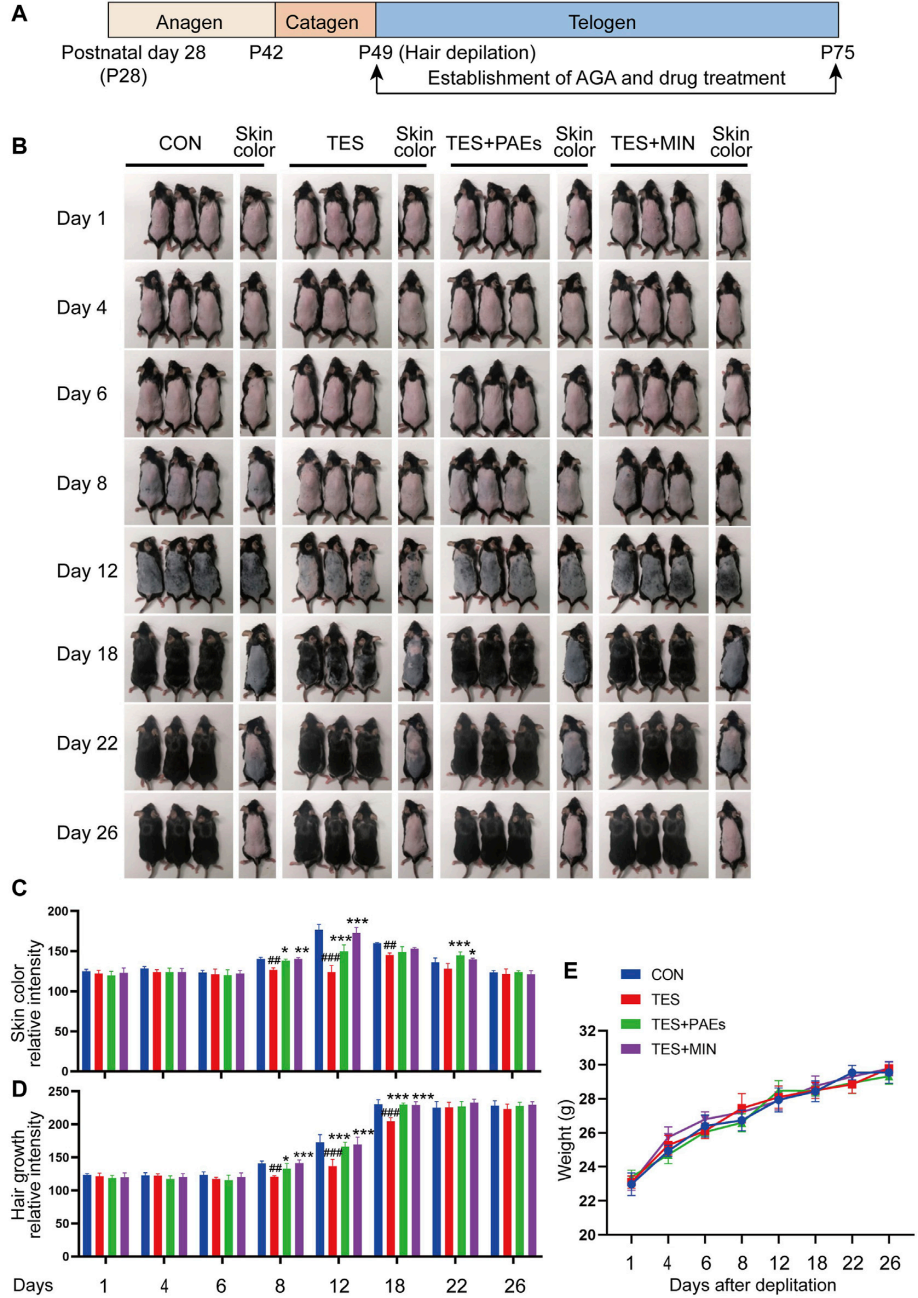
PAEs can promote hair regrowth by activating the anagen and delaying catagen in AGA mice. **(A)** Scheme of the HF cycle of postnatal mouse and timeline of AGA development and PAEs treatment. **(B)** Representative skin color and hair regrowth status of each group on days 1, 4, 6, 8, 12, 18, 22, and 26 (Note: In order to better reflect the relation between skin color and hair cycle, one mouse’s skin color was chosen for presentation. The mice’s dorsal hair is non-depilated on days 1, 4, 6, 8, and 12, and depilated on days 18, 22, and 26 because the mice’s hair growth will cover the skin color at these time points). **(C–D)** Quantification of skin color and hair growth with Image J. **(E)** The changes in body weight at different time points. Comparing to CON group, ^##^ *p* < 0.01, ^###^ *p* < 0.001, and the TES group, ^*^ *p* < 0.05, ^**^ *p* < 0.01, ^***^ *p* < 0.001.

We then observed a complete hair cycle of hair growth in AGA mice. On postnatal day 49, 8 mg/mL PAEs gel was applied for 26 days in a row to test the change of PAEs in AGA mice’s hair cycle (Figure 1A). Skin color could be a biomarker to determine the stage of the HF cycle because the activation of the new anagen phase was indicated by a considerable darkening due to pigmentation in HFs during the anagen phase (Plikus and Chuong, 2008). As illustrated in Figure 1B, the skin color in PAEs group changed from pink to gray at day 8, suggesting the mice began to enter the anagen. In contrast, the TES group remained pink. On day 12, the PAEs-treated group’s skin color was noticeably darker than that of the TES group, and nearly identical to the MIN group. On day 18, the PAEs group’s mice skin tone darkened to a greater extent than that of the AGA mice. From day 18 to day 22, the mice’s skin color started to change from dark to gray and pink to different extents, suggesting the mice began to enter the catagen, while the variability of PAEs group was slighter than the other three groups. In addition, the same trend also applies to hair density (Figures 1C, D). On day 26, almost all mice are restored to telogen. Meanwhile, each group showed the same trend of body weight changes under different time points (Figure 1E), indicating topical administration is harmless to mice. These findings indicated that PAEs could stimulate hair regrowth by accelerating the anagen and delaying catagen in AGA mice.

### 3.2 PAEs alleviated the shortening of anagen period, skin thinness, reduction of hair bulb size, HF length, HF number, and HF regression in AGA mice

**FIGURE 2:**
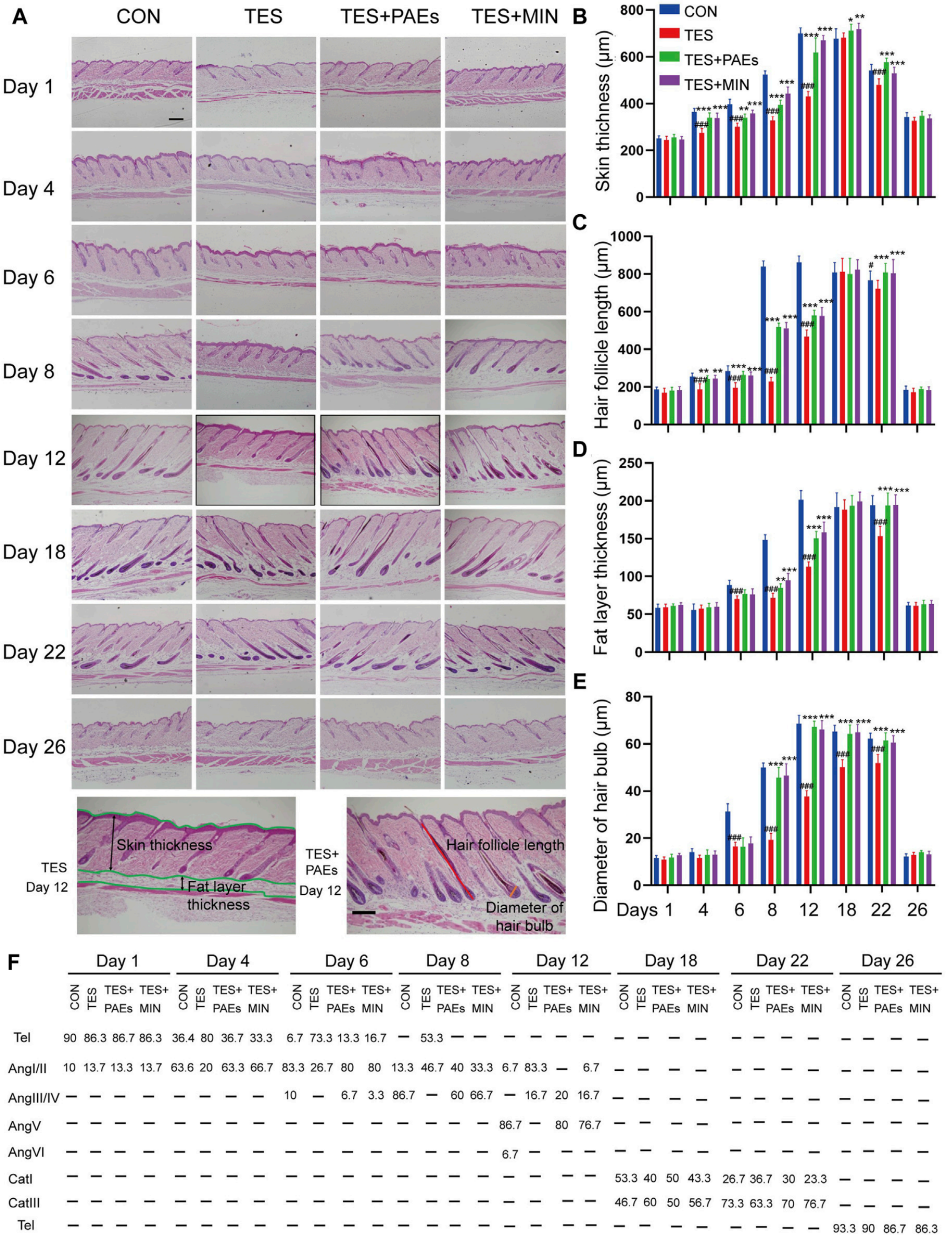
PAEs facilitated the cycle of HF regeneration and alleviated hair thinning in AGA mice. **(A)** Representative longitudinal sections of mouse skin of each group stained with H&E on days 1, 4, 6, 8, 12, 18, 22, and 26. The last row contains two H&E images of the TES + PAEs group and the TES group at day 12. Lines of different colors indicate the positions of the four measured parameters: hair follicle length, hair bulb diameter, skin thickness, and fat layer thickness. Scale bar: 200 µm. **(B–E)** Effects of PAEs on the thickness of skin and fat layer, HF’s length, and hair bulb’s diameter in AGA mice. **(F)** Quantification of each group’s percentage of HFs at the designated hair cycle phases (n = 30 HFs). Comparing the CON group, ^##^ *p* < 0.01, ^###^ *p* < 0.001, and the TES group, ^*^ *p* < 0.05, ^**^ *p* < 0.01, ^***^ *p* < 0.001.

To investigate the histological changes of AGA mice after PAEs treatment, dorsal skin was collected on days 1, 4, 6, 8, 12, 18, 22, and 26, and examined histologically (Figure 2A). Five indicators were assessed (Figures 2B–F): the skin and fat layer’s thickness, the HFs’ length, the hair bulb’s diameter, and the percentage of HFs in the specified hair cycle phases. Based on the previously described classification, the hair cycle progression can be analyzed, on days 4, 6, 8, and 12, the HF in TES + PAEs and CON groups was more than those in the TES group. Additionally, AGA mice had earlier regression from day 18 to day 22 in comparison to the other groups. The histological pathology data also supported the notion that PAEs regulated the hair cycle by activating the anagen and delaying the entry of catagen in AGA mice. Furthermore, when compared to the CON and PAEs-treated groups, the AGA mice at these periods exhibited considerably thinner skin and fat layers as well as smaller hair bulbs. Taken together, these findings indicate that PAEs regulates the hair cycle by alleviating TES-induced thinner skin and fat layer, hair bulb diminishes, and HF shortened in AGA mice.

We also estimated the effect of HF counts after PAEs treatment in AGA mice, The changes in the HF number of each group in transverse sections were assessed on days 1, 4, 6, 8, 12, 18, 22, and 26. It is apparent that PAEs raised the HF numbers in comparison to those in AGA mice (Supplementary Figures S2A, B). Furthermore, it has been reported that the hair morphogenesis in AGA mice can be affected, leading to a thinning of the hair shafts (Fu et al., 2021). We also found that the ratio of the hair shaft diameter of guard hair to the inner root sheath (IRS) diameter increased in the TES + PAEs group than the TES group (Supplementary Figures S1C, D), indicating PAEs alleviated the hair thinner in AGA mice.

### 3.3 PAEs regulated the hair regeneration process and alleviated the morphological changes of four hair subtypes in AGA mice

**FIGURE 3:**
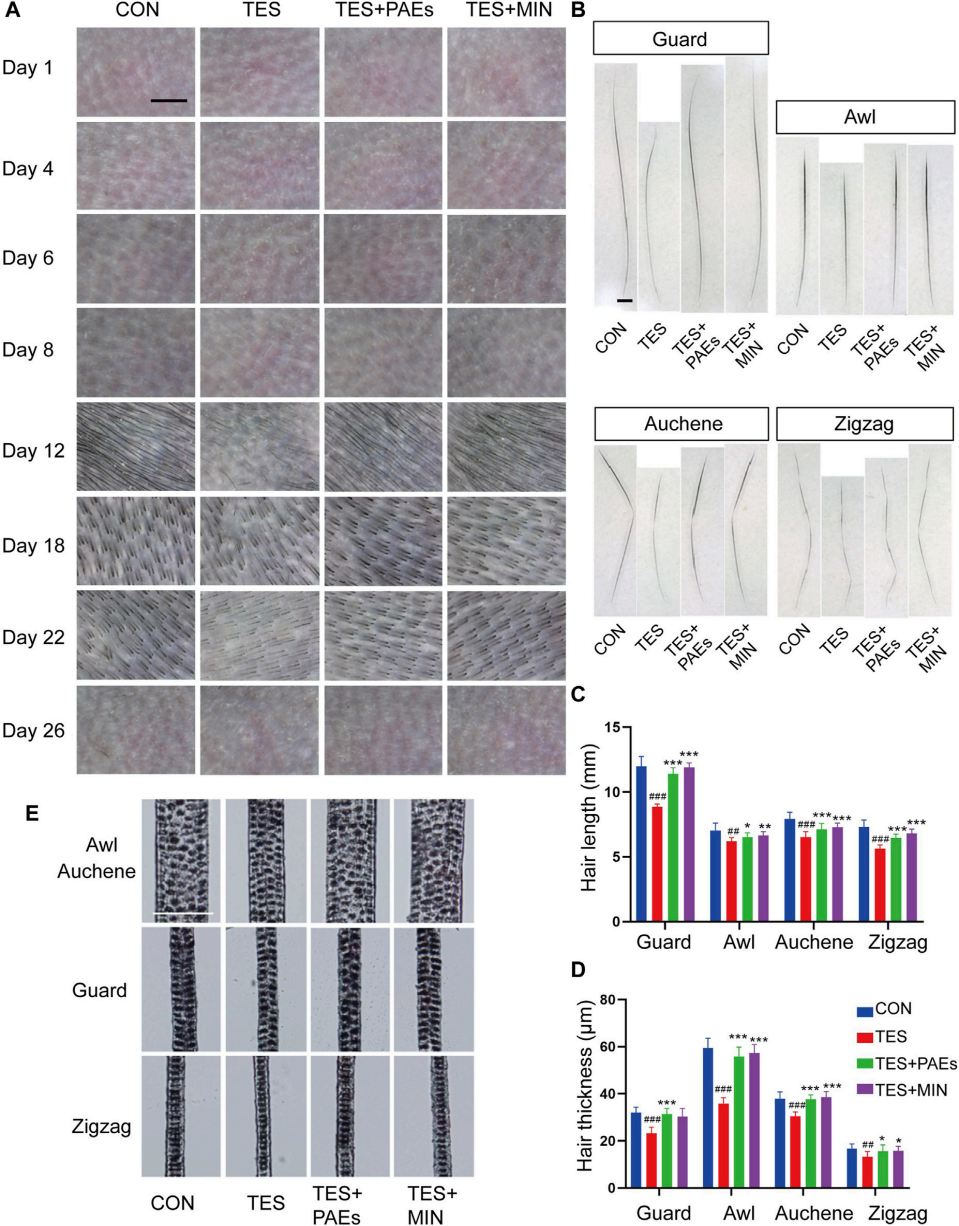
PAEs regulated the process of hair regeneration, mitigating the alterations in HF type, thickness, and length in AGA mice. **(A)** Representative photos of MED of each group on days 1, 4, 6, 8, 12, 18, 22, and 26. Scale bar: 1 mm. **(B)** Pictures of four subtypes’ hair on day 26. Scale bar: 50 μm. **(C–D)** Analysis of four dorsal HF subtypes’ thickness and length. **(E)** Four hair subtypes’ morphologies for each group on day 26. Comparing the CON group, ^##^ *p* < 0.01, ^###^ *p* < 0.001, and the TES group, ^*^ *p* < 0.05, ^**^ *p* < 0.01, ^***^ *p* < 0.001.

To confirm our findings and validate our theory, the MED was used to observe the micro-characteristic changes of dorsal hair, each hair subtype’s morphology, and hair shaft length in each group. From the images captured by MED, it’s clearly found that the PAEs could alleviate the reduction in number and thickness in AGA mice, especially on days 12, 18, and 22 (Figure 3A). Furthermore, skin color observed from the MED images reflects the variation of the hair cycle, which also supported our above findings to some extent.

Additionally, the mouse hair coat is made up of four HF subtypes (guard, awl, auchene, and zigzag). To explore how PAEs affect hair substyles in AGA mice, the dorsal hair was collected on day 26. The shorter hair shafts of AGA mice are evident, particularly in the guard and auchene hair (Figures 3B, C). Moreover, in four groups presented with rows of medulla cells, the thickness of all subtypes was markedly thinner morphologically in AGA mice (Figures 3D, E). These findings suggest that PAEs regulates the process of hair regeneration, hence mitigating the variations of four subtypes’ hair in AGA mice.

### 3.4 PAEs stimulated HFSCs, ORS cells, and hair bulb cell proliferation in AGA mice

**FIGURE 4:**
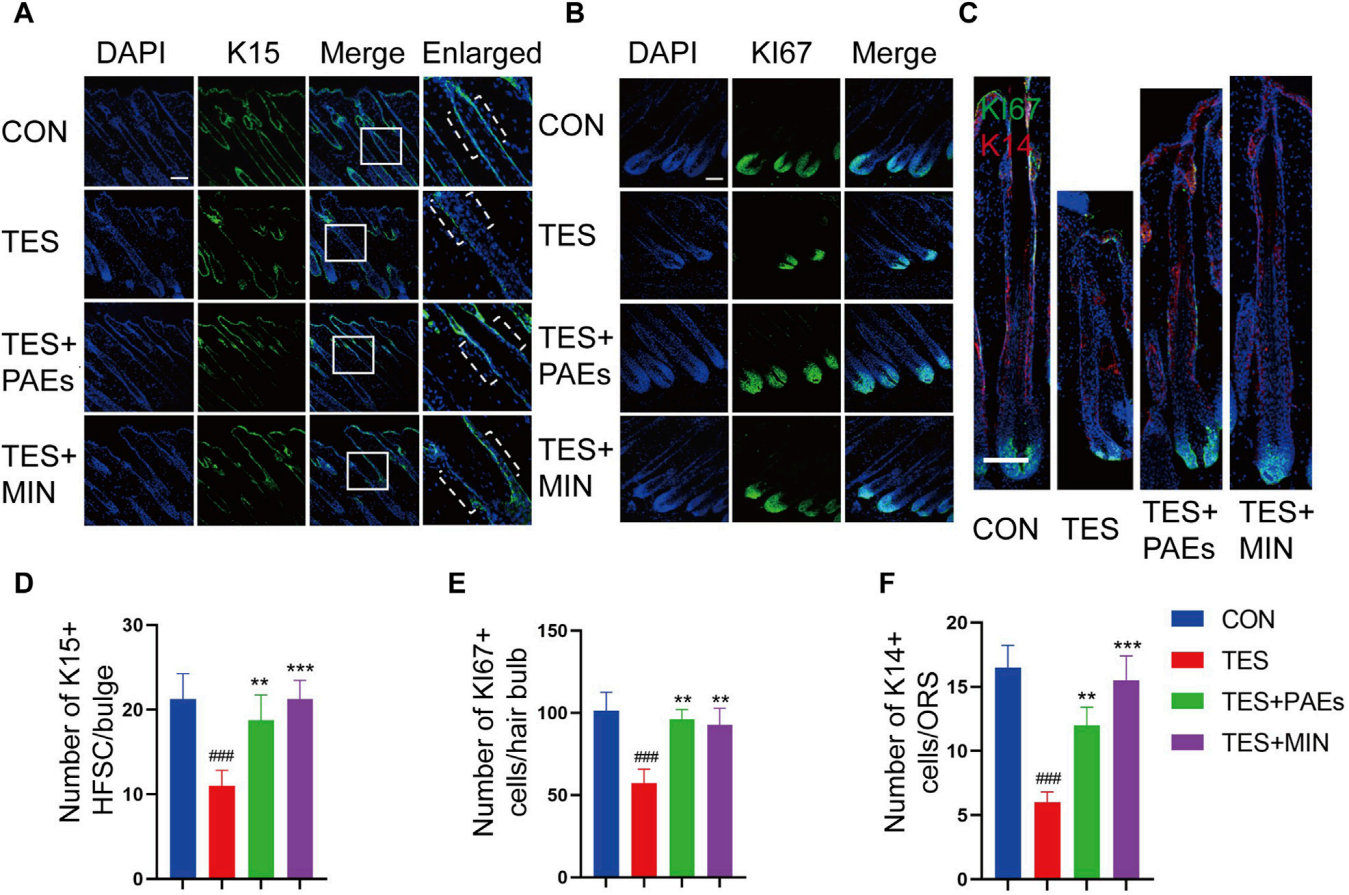
Immunofluorescence images of HFSC **(A)**, hair bulb cells **(B)**, and ORS cells **(C)** of dorsal skin in each group. The dashed area in **(A)** indicates the location of the HF bulge. Scale bar: 100 μm. Quantitative evaluation of localized populations of K14 **(F)**, Ki67 **(E)**, and K15 **(D)** cells. (n = 3 per group). Comparing the CON group, ^###^ *p* < 0.001, and the TES group, ^**^ *p* < 0.01, ^***^ *p* < 0.001.

The process of hair formation and morphogenesis (such as thickness and length) requires the participation of multiple HF types of cells (Saxena et al., 2019). HFSC plays an important role in HF regeneration and hair cycle regulation, its interaction with hair bulb cells and ORS cells is also crucial for the formation of HF structure (Fuchs, 2007). In this study, we used K15 (Figure 4A) as the HFSC marker in the bulge of HF, Ki67 (Figure 4B) as the cell proliferation marker in the hair bulb, and K14 (Figure 4C) as the cell marker in the ORS (Coulombe et al., 1989; Inoue et al., 2009). The immunofluorescence staining results of mouse skin at day 12 indicated that the count of Ki67^+^, K15^+^, and K14^+^ cells was increased in the PAEs group compared to AGA mice (Figures 4D–F). Taken together, these findings reveal that PAEs reverses the decrease in HFSCs, ORS cells, and hair bulb cells in AGA mice.

### 3.5 Combined network pharmacology and PAEs transcriptomics to predict the possible targets and signaling pathways of PAEs for AGA treatment

Network pharmacology integrates information from public databases to analyze the overall mechanisms of drug action and predict its effects on diseases. Since PAEs have the effect of promoting hair growth in AGA mice, we conducted *de novo* transcriptomic analysis of pilose antler, and combined with AGA disease targets from public databases, predicted the target genes of pilose antler for treating AGA.

**FIGURE 5:**
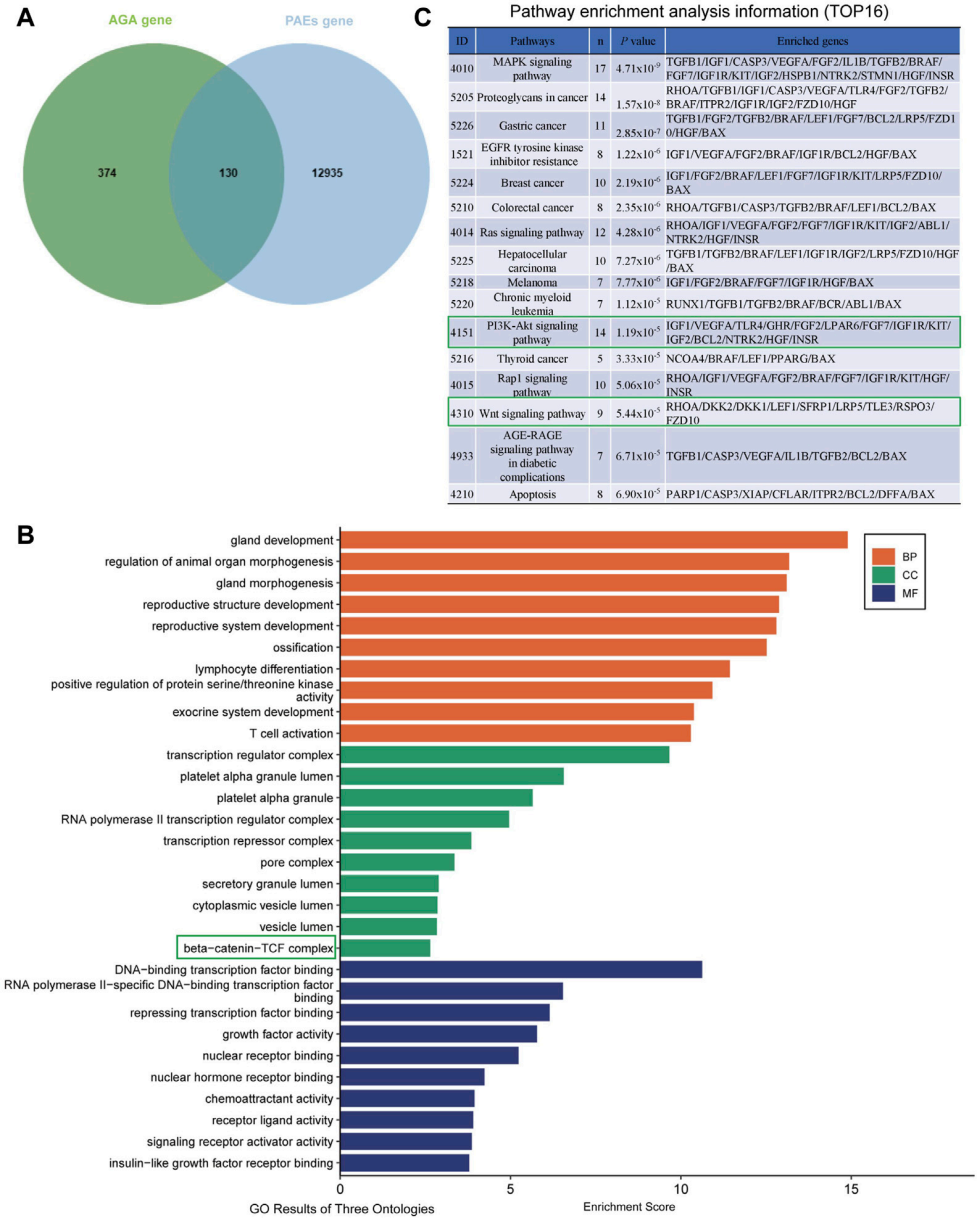
Prediction of possible targets and signaling pathways of PAEs for AGA treatment. **(A)** Intersection of PAEs active ingredient targets with AGA disease targets. **(B)** GO enrichment analysis of intersecting targets (TOP 10 in GO results of three ontologies). **(C)** Pathway enrichment analysis information (TOP16).

The analysis results indicated that a total of 13065 component targets were collected from PAEs transcriptomics data, and then 504 AGA-related targets were collected from the GeneCards database and DisGeNET database. Utilizing the jvenn method, we identified the intersection of disease-related targets and PAEs targets, resulting in 130 potential targets for PAEs in treating AGA (Figure 5A). Subsequently, these 130 core targets underwent GO and KEGG enrichment analysis.

From the KEGG analysis, we can see some growth factors such as transforming growth factor-β1/2 (TGF-β), IGF1, VEGF, FGF2/7, and HGF, suggesting that the therapeutic effect of PAEs on AGA may be related to the function of the above growth factors (Supplementary Figures S3).

From the cellular component (CC) section of the GO analysis entries, it can be seen that the β-Catenin-TCF complex plays a significant role in biological regulation (Figure 5B). Meanwhile, The KEGG pathway analysis revealed the top 16 enriched signaling pathways, prominently including MAPK, Ras, PI3K-Akt, and Wnt signaling pathways (Figure 5C). Among the four signaling pathways, it has been shown that MAPK (Akilli Ozturk et al., 2015), PI3K-AKT (Liu et al., 2021) and Wnt signaling pathways (Veltri et al., 2022) play important roles in the regulation of hair growth and growth cycle. The original KEGG pathway maps for Wnt-Catenin and the PI3K-AKT pathways were in Supplementary Figures S4A, B. Considering that the PI3K-AKT, and Wnt signaling pathways are interconnected by GSK3β, we then tested whether these two signaling pathways were altered when investigating the mechanism of PAEs promoting hair growth in AGA mice, aiming to attempt to verify this potential mechanism.

### 3.6 PAEs activated the Wnt/β-catenin pathway in AGA mice

**FIGURE 6:**
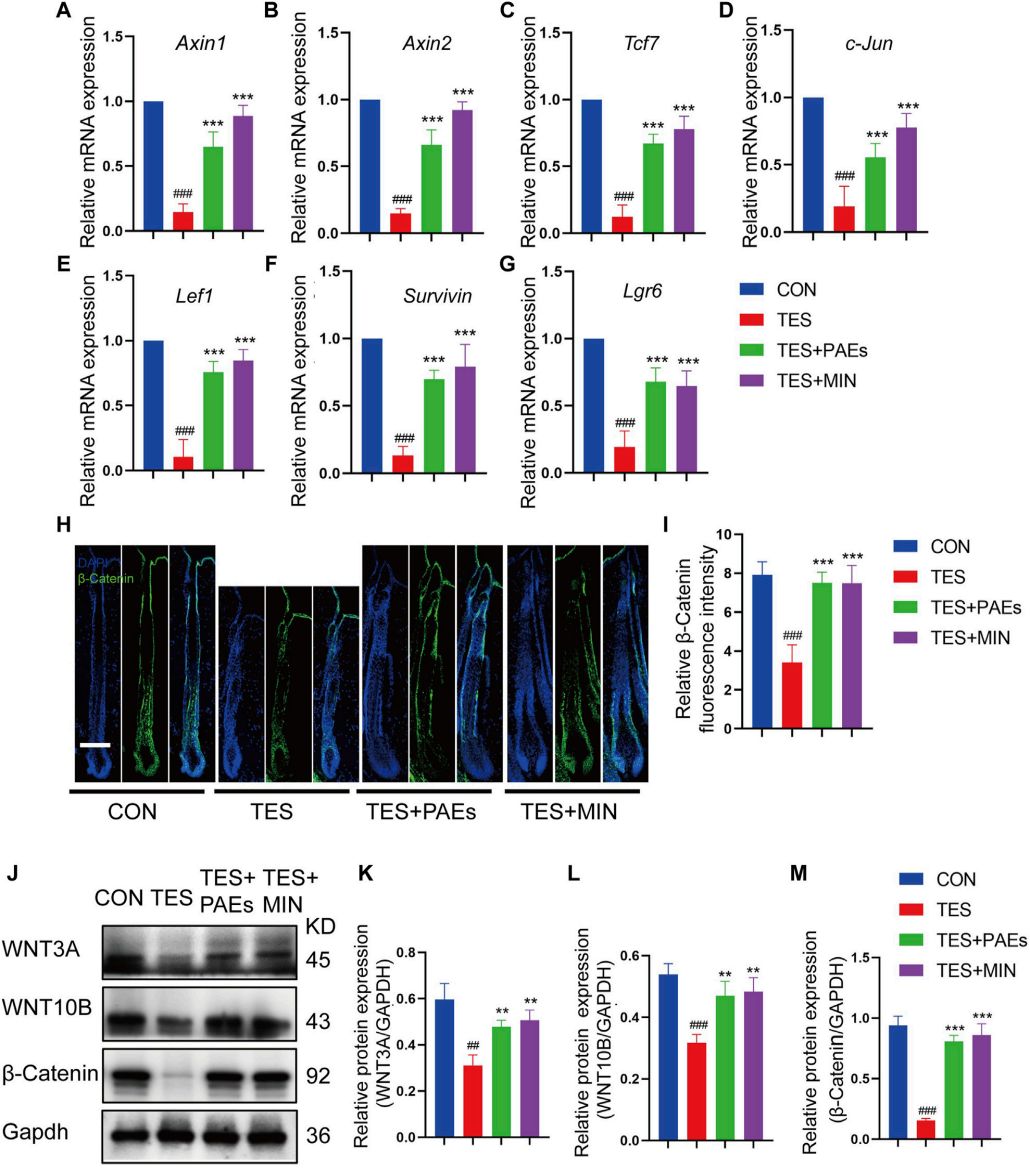
PAEs upregulated the Wnt/β-catenin pathway in AGA mice. The gene expression of **(A)** *Axin1*, **(B)** *Axin2*, **(C)** *Tcf7*, **(D)** *c-Jun*, **(E)** *Lef1*, **(F)** *Lgr6*, and **(G)** *Survivin* were measured following Gapdh normalization. **(H)** Representative immunofluorescence images of β-catenin in four groups. **(I)** Quantitative fluorescence analysis of the β-catenin in HF. **(J)** The protein bands of WNT3A, WNT10B, and β-catenin in each group. Gapdh served as the loading control. **(K)** WNT3A, **(L)** WNT10B, and **(M)** β-catenin band intensities were measured and standardized to Gapdh expression using Image J. Comparing the CON group, ^##^ *p* < 0.01, ^###^ *p* < 0.001, and the TES group, ^**^ *p* < 0.01, ^***^ *p* < 0.001.

The Wnt/β-catenin pathway plays a critical role in HF development (Deschene et al., 2014; Jin et al., 2021). To determine if PAEs might affect this pathway, the mRNA level of up-and downstream genes of Wnt/β-catenin was examined, including *Axin1*, *Axin2*, *Lef1*, *Tcf7*, *c-Jun*, *Lgr6*, and *Survivin* (Figures 6A–G), and protein level of β-catenin (Figures 6H–J, M) and its two ligands (WNT3A and WNT10B) (Figures 6J–L) in back skin of each group. It was found that the above-mentioned genes and the proteins of β-catenin, WNT3A, and WNT10B were dramatically upregulated by PAEs in AGA mice, suggesting that this pathway could be triggered by PAEs.

### 3.7 PAEs triggered the PI3K/AKT pathway to stabilize β-catenin in AGA mice

**FIGURE 7:**
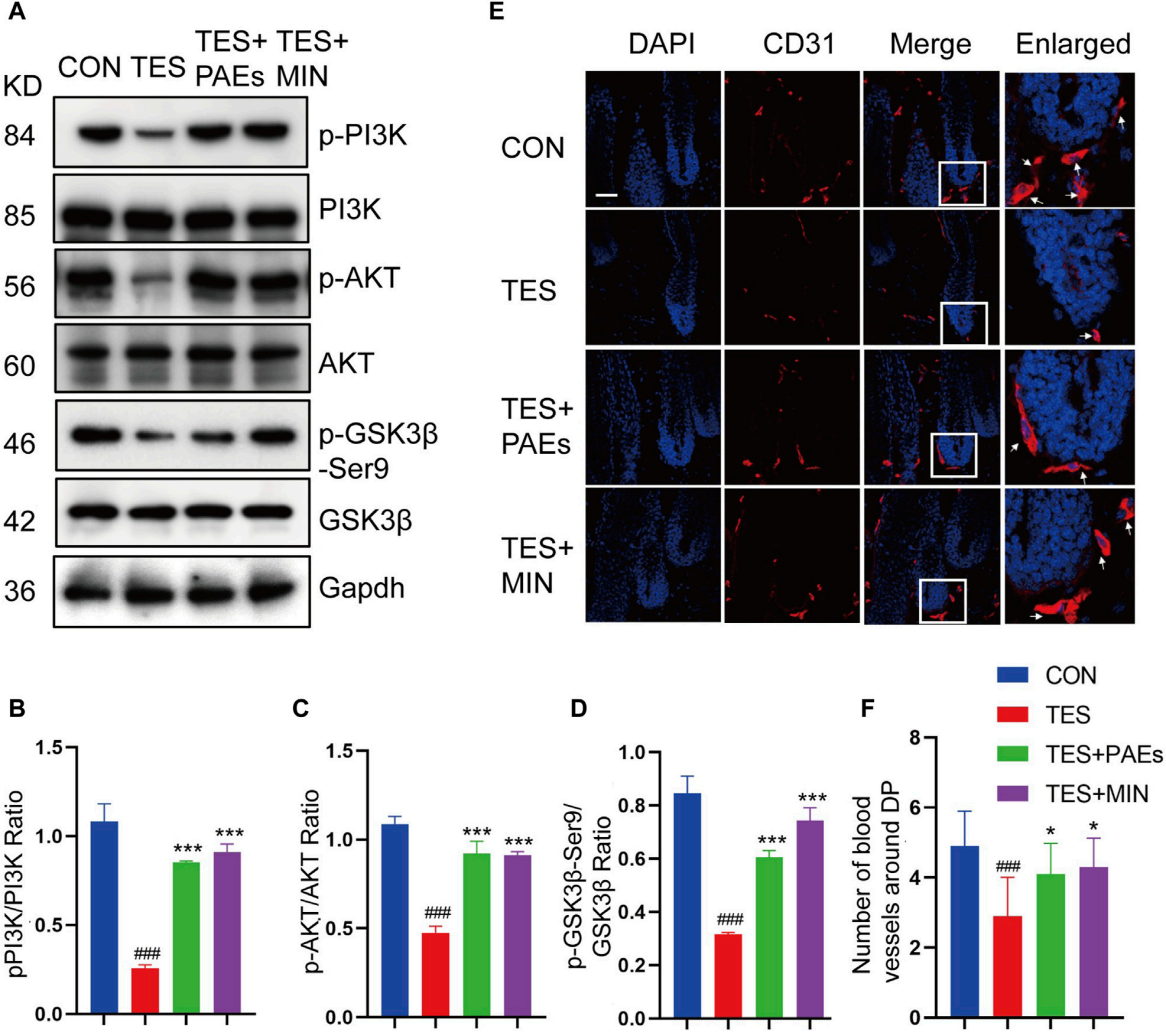
PAEs also stimulated hair growth by triggering the PI3K/AKT pathway in AGA mice. **(A–D)** The level of related proteins in the PI3K/AKT pathway. Gapdh served as the loading control. ImageJ was used to quantify the band intensities. **(E)** Immunofluorescence images of CD31 vessel marker (arrow position) around HF in each group. The white box indicates the blood vessels surrounding the DP. **(F)** Quantitative analysis of the quantity of vessels around DP (*n* = 3). Comparing the CON group, ^###^ *p* < 0.001, and the TES group, ^*^ *p* < 0.05, ^***^ *p* < 0.001.

GSK-3β could adversely regulate β-catenin, and the inhibitory phosphorylation site GSK-3β at Ser9 could be stimulated by AKT and then induce a cascade reaction to produce β-catenin (Zhang et al., 2019). Meanwhile, the stimulation of the PI3K/AKT pathway is necessary for new HF generation which is mediated by skin-derived precursors and epidermal stem cells (Chen et al., 2020). Therefore, the p-PI3K, p-AKT, and p-GKS-3β-Ser9 protein levels were tested in AGA mice after PAEs treatment. As illustrated in Figures 7A–D, PAEs reversed the suppression of these protein levels significantly. This suggests that PAEs may have a promoting effect on the accumulation of β-Catenin by activating the PI3K-AKT pathway. Furthermore, the PI3K/AKT pathway plays an essential role in angiogenesis (Karar and Maity, 2011). The new blood vessels surrounding DP provide nutrients to support hair growth, Studies have reported that under the influence of androgen receptors, the blood vessels surrounding the DP degenerate, resulting in a decrease in their number (Deng et al., 2022). We found the PAEs perhaps increased the number of blood vessels around the DP (Figures 7E, F) in AGA mice.

### 3.8 PAEs could promote HFSC proliferation and activate the AKT and Wnt pathways

**FIGURE 8:**
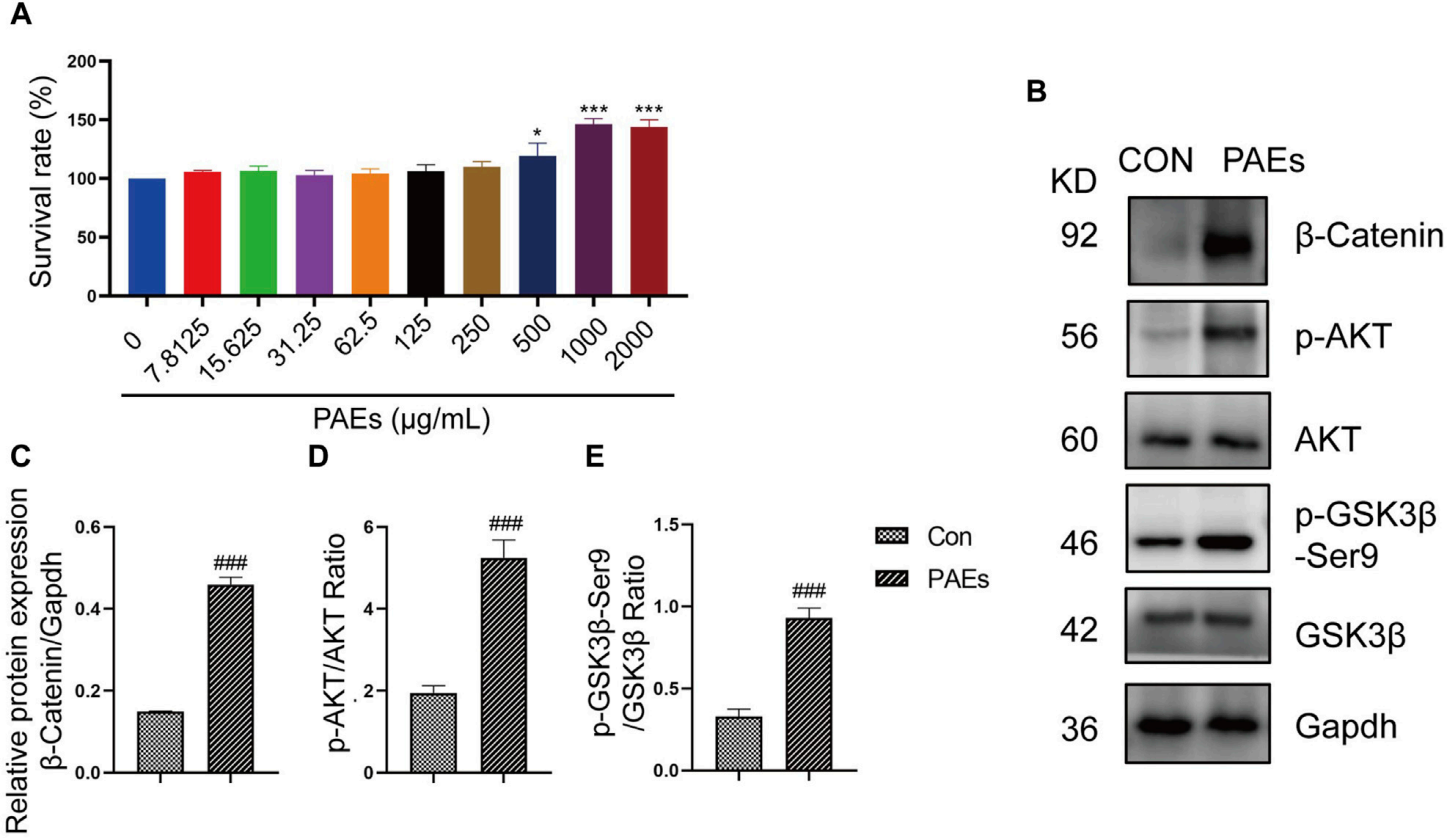
PAEs activated the HFSC proliferation by upregulating the expression of the AKT and Wnt pathways. **(A)** Statistics of proliferation of HFSC by PAEs treatment at 10 different drug concentrations (0–2000 μg/mL) (n = 6). **(B–E)** The protein level and statistical graphs of p-AKT, p-GSK3β-Ser9, and β-Catenin in AKT and Wnt pathways in HFSC after PAEs treatment. Gapdh served as the loading control. Comparing the CON group, ^###^ *p* < 0.001.

The detection of protein and gene changes related to both the PI3K-AKT and Wnt-β-Catenin pathways in AGA mice validated our speculation, suggesting that the treatment of PAEs in AGA mice is closely related to the AKT and Wnt pathways. Then We also examined the link between the activation and proliferation of HFSC *in vitro* and the AKT and wnt pathways after PAEs treatment. HFSC is closely related to the regulation of the HF cycle and the promotion of the formation of HF structures such as the hair shaft and the IRS (Lin et al., 2020). Meanwhile, the AKT and Wnt pathways have an important role in promoting the proliferation and differentiation of epidermal stem cells and hair regrowth (Wang et al., 2017). We found that PAEs at 250–1000 μg/mL had a significant pro-proliferative effect on HFSC, with a proliferation rate of 150.4% at 1000 μg/mL (Figure 8A). In addition, the protein level of p-AKT, p-GSK3β-Ser9, and β-Catenin was obviously increased in HFSC after PAEs treatment (Figures 8B–E).

### 3.9 PAEs stimulated the HFSC proliferation by activating the AKT and Wnt pathways

**FIGURE 9:**
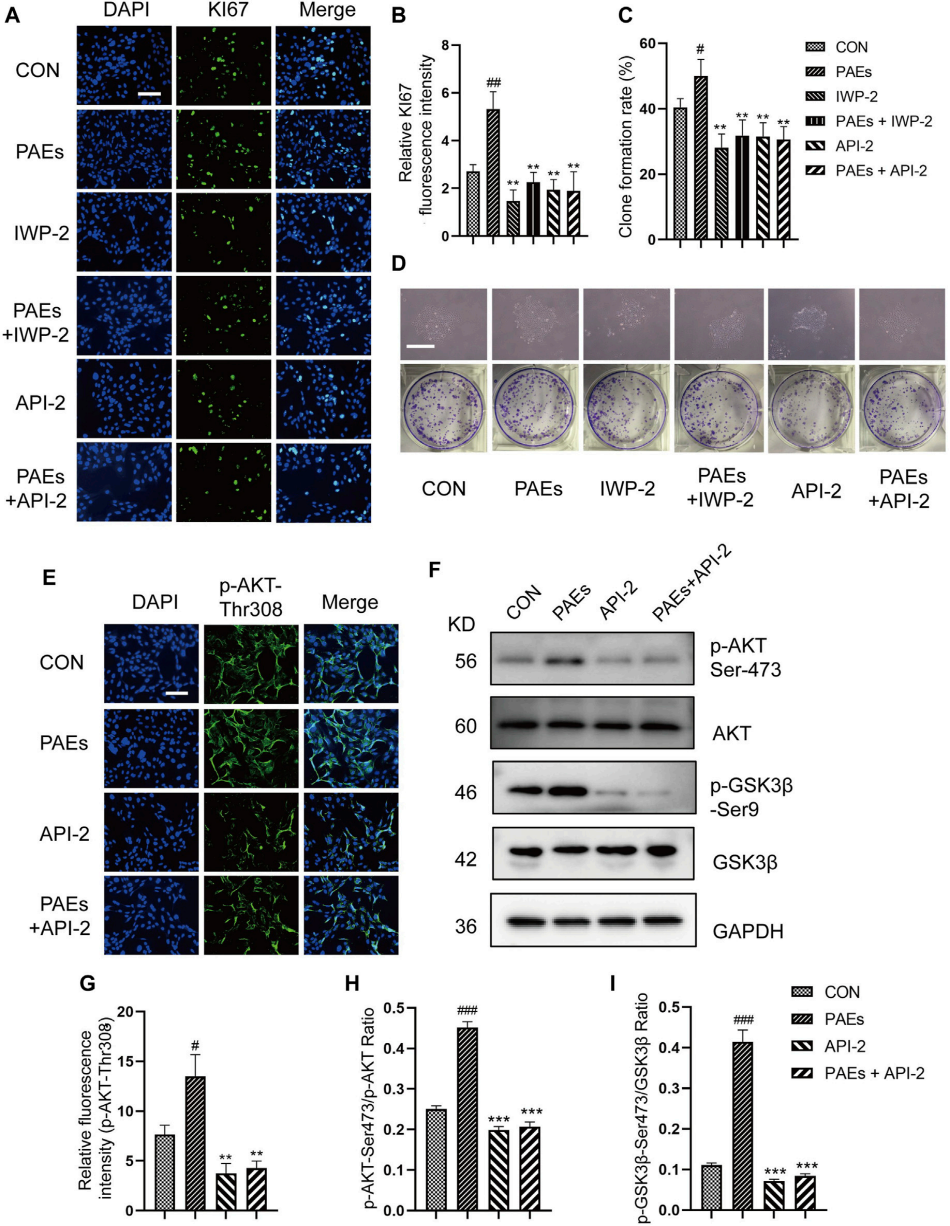
PAEs stimulated the proliferation of HFSC by activating the AKT and Wnt pathways. **(A,B)** Representative images of immunofluorescence and statistical analysis of KI67 in each group after AKT and Wnt pathway blockade. Scale bar: 200 μm. **(C,D)** Statistical analysis of representative images and clone formation rate of HFSC clone formation experiments in each group after AKT and Wnt pathway blockade. Scale bar: 500 μm. **(E)** Representative images of immunofluorescence of p-AKT-Thr308 in each group after AKT pathway blockade in HFSC, Scale bar: 200 μm, n = 3. **(F)** Representative Western blot images of p-AKT-Ser473 and p-GSK3β-Ser 9 of each group in HFSC. **(G–I)** Statistical analysis of immunofluorescence and Western blot images. Comparing the CON group, ^###^ *p* < 0.001, ^#^ *p* < 0.05, and the PAEs group, ^*^ *p* < 0.05, ^**^ *p* < 0.01, ^***^ *p* < 0.001.

To verify whether the activation of PAEs on HFSC is mediated through the AKT and Wnt pathways. We first detected the proliferative effect of HFSC after blocking the AKT activity and Wnt pathway. API-2 is a specific AKT inhibitor, mainly by suppressing the AKT phosphorylation at activation sites (Thr308 and Ser473). IWP-2 is an inhibitor of Wnt pathway. From the results of immunofluorescence of KI67 (Figures 9A, B) and clone formation experiments (Figures 9C, D), it can be seen that the proliferation and clone formation ability of HFSC was significantly enhanced after PAEs treatment. However, this effect was significantly reversed after blocking AKT activity and the Wnt pathway in HFSC. And the proliferative activity of HFSC in the PAEs + IWP-2/API-2 group could not be restored. It was suggested that the promotion of HFSC proliferation by PAEs might be closely related to the activation of the AKT and Wnt pathways.

To further validate our conjecture, we intervened with PAEs in HFSC that blocked AKT activity and Wnt pathway, and detected the level of relevant proteins and genes in the AKT and Wnt pathways. As can be seen from the results of immunofluorescence (Figures 9E, G) and Western blot (Figures 9F, H, I), the protein levels of p-AKT at both sites (Thr308 and Ser473) and p-GSK3β-Ser9 showed an obvious trend to increase after PAEs treatment in HFSC. However, the protein levels of p-AKT and p-GSK3β-Ser9 decreased significantly after blocking the AKT activity and did not recover after PAEs intervention. We also tested the mRNA levels of up-and downstream genes in the Wnt pathway, such as *Survivin*, *LEF1*, *Axin1*, *Axin2*, *Lgr6*, *Tcf7*, and *Ccnd1* (Supplementary Figures S5A) and the levels of β-Catenin, WNT3A and WNT10B (Supplementary Figures S5B–G), then found these gene expressions increased in HFSC after PAEs treatment. However, the expressions of these genes were obviously suppressed after blocking the Wnt pathway, and the expressions did not recover under the PAE treatment. These results showed that the proliferation of HFSC induced by PAEs may be closely related to the activation of the AKT and Wnt pathways.

## 4 Discussion

AGA is a progressive disease that lacks effective treatment, its incidence tends to increase with age (Lolli et al., 2017). Finasteride and topical minoxidil (MIN) have demonstrated limited efficacy alongside undesirable adverse effects. Consequently, it is imperative to investigate alternate therapy approaches for AGA. Robust evidence underscores the efficacy of TCM in fostering hair growth (Zhou et al., 2020; Gao et al., 2023). Pilose antler has exhibited promising potential for hair growth promotion in recent studies (Yang et al., 2012; Li et al., 2014; Lee et al., 2016; Tansathien et al., 2021). We present here the first evidence that PAEs could expedite the hair growth cycle, mitigate hair morphological changes, enhance the proliferation of cells associated with HF growth, and increase HF number in AGA mice, the mechanism of which may have a close relation with the activation of the AKT/β-Catenin pathway. Meanwhile, the proliferation of HFSC *in vitro* after PAEs treatment was associated with AKT/β-Catenin pathway.

Firstly, we clarified the pharmacological efficacy of PAEs to promote hair growth in AGA mice and determined the optimal drug concentration. In the next study, we observed the effects of PAEs on the hair cycle, and hair morphology in AGA mice during a complete hair cycle. The hair growth ability of PAEs was assessed in AGA mice by observing skin color and hair density at various time points. Skin color changes serve as an indicator of the HF cycling stage (Plikus and Chuong, 2008), with our findings indicating that the PAEs group exhibited an earlier transition from pink to gray and displayed the lightest change from black to pink compared to the TES group. Concurrently, a similar trend was observed in hair density, revealing that PAEs could expedite anagen activation and delay the anagen-catagen transition in AGA mice. Prominent characteristics of AGA include shortened hair, reduced HF number, and progressive miniaturization (Premanand and Reena Rajkumari, 2018), primarily attributed to the depletion and functional decline of the HFSCs (Lei and Chuong, 2016). As to be expected, the morphology of hair in this study is significantly consistent with the AGA model. Histological analysis of longitudinal and transverse sections at different time points suggested that HF in the PAEs group exhibited significantly longer and thicker, had more proliferating cells in the hair bulb, and increased more hairs than the TES group. Furthermore, Adipose precursor cells are both required and sufficient to trigger the activation of HFSCs during the hair-growth process (Festa et al., 2011), and the TES has been reported to promote lipolysis while suppressing preadipocyte proliferation and adipocyte development (O'Reilly et al., 2014). We observed that compared with the CON group, the skin and adipose layer of AGA mice were significantly thinner, and found that PAEs could improve this condition, possibly by alleviating the inhibitory effect of TES on adipose layer development.

Some additional types of cells in HF are closely linked to the periodic activation or quiescence of HFSCs and are subject to strict regulation (Fuchs, 2007; Driskell et al., 2011). In AGA mice, the imbalance between quiescence and activation signals leads to a failure or delay in initiating a new hair cycle for HFSCs (Fu et al., 2021). The interaction of HFSC with other cells in the HF is crucial for the formation of hair follicle structure. Our study showed that TES induced a decrease in HFSCs, ORS cells, and hair bulb cells, making it difficult to stimulate hair growth, but this effect was reversed by PAEs significantly. The mouse hair coat is made up of four HF subtypes. Guard hairs, which have the longest hair shafts, are characterized by two rows of medulla cells and a lack of kinks. Awl hairs are shorter and straighter, which have one or two more rows of cells than guard hairs. Auchene hairs are similar to awl hairs in development and morphology, but they have a single kink in the hair. Zigzag hairs, the most abundant type are characterized by more than two kinks and a line of medullary cells (Cui et al., 2010; Li and Ginty, 2014). It has been claimed that following dihydrotestosterone treatment, the length and thickness of all four hair types were noticeably shorter and thinner (Fu et al., 2021). The development of hair requires the involvement of various hair follicle cells, including HFSC, hair bulb cells, and ORS cells (Lei et al., 2014; Houschyar et al., 2020; Xie et al., 2022). Our research found PAEs could improve the formation of HF structure by activating the proliferation of HFSC, ORS cells, and hair bulb cells in AGA mice.

From the above data in this study, the PAEs could alleviate the morphological changes and increase the number of HF cells related to hair development in AGA mice. Then, we further studied the molecular mechanism of PAEs in hair promotion ability. We integrated the results of network pharmacology and transcriptomics analysis of pilose antler to show that the active ingredients in PAEs for treating AGA are mainly some growth factors, such as TGF-β, IGF1, VEGF, FGF, and HGF. Meanwhile, among the KEGG-enriched pathways, both the PI3K-AKT and Wnt pathways cross-link with GSK3β, and thus the changes in both pathways in AGA mice by PAEs were detected in this study.

The β-catenin could impact the regeneration and shape of HF (Enshell-Seijffers et al., 2010). Wnt/β-catenin signals are frequently reduced in AGA (Leiros et al., 2017). Our findings found that the upregulation of WNT3A and WNT10B could trigger cascade reactions and cause β-catenin stabilization and accumulation after PAEs treatment in AGA mice. Additionally, PAEs upregulated the level of phosphorylated AKT, PI3K, and the inhibitory phosphorylated GSK-3β-Ser9, along with other correlated targets in the Wnt/β-catenin pathway including the genes of *Axin1*, *Axin2*, *Lef1*, *Tcf7*, *c-Jun*, *Lgr6*, and *Survivin*. Moreover, the control of angiogenesis is tightly linked to the PI3K/AKT pathway (Karar and Maity, 2011). Meanwhile, the AGA could cause the regression of blood vessels surrounding the DP (Deng et al., 2022). Our results revealed that PAEs perhaps increase the number of vessels around the DP to support sufficient nutrients for hair growth. We will conduct additional experiments to further validate this hypothesis in subsequent studies.

HFSC plays an important role in the neogenesis of HF and the regulation of the hair cycle (Li et al., 2020). *In- vitro* studies showed that PAEs had a significant pro-proliferative effect on HFSC and were able to upregulate the protein level of p-AKT, p-GSK3β-Ser9, and β-catenin. After blocking the Wnt pathway and AKT activity in HFSC, the proliferative activity of HFSC was inhibited, and the level of the relevant proteins and genes in the AKT and Wnt pathways were significantly reduced, and none of them were recovered after the PAEs treatment. This suggested that the proliferation of HFSC by PAEs is achieved by stimulating the AKT and Wnt pathways.

In this paper, we confirmed that PAEs has the ability to promote hair growth in AGA mice and stimulate the proliferation of HFSC *in vitro*. We hypothesized that it is related to many types of growth factors contained in pilose antler. These findings could potentially contribute to the development of therapeutic compounds from pilose antler to enhance hair regrowth and lay the foundation that directs future efforts to find novel medications that will effectively treat AGA.

## 5 Conclusion

The hair growth-promoting effect of PAEs in AGA mice may be closely related to the stimulation of the AKT and Wnt pathways, which in turn activates the function of HFSC.

## Data Availability

The raw data supporting the conclusions of this article will be made available by the authors, without undue reservation.

## Abbreviations

AGA, Androgenetic alopecia; CCK8, Cell counting kit 8; DP, Derma papilla; FGF, Fibroblast growth factor; HF, Hair follicle; HFSC, Hair follicle stem cell; H&E, Hematoxylin and eosin; HGF, Hepatocyte growth factor; IRS, Inner root sheath; MED, Medical electronic dermoscopy; MIN, Minoxidil; ORS, Outer root sheath; PAEs, Pilose antler extracts; RT-qPCR, Real-time reverse transcription-quantitative polymerase chain reaction; TES, Testosterone; VEGF, Vascular endothelial growth factor.
