# Supplementary material for: Pilose antler extracts promotes hair growth in androgenetic alopecia mice by activating hair follicle stem cells via the AKT and Wnt pathways
Source: Front Pharmacol. 2024 Jul 9;15:1410810. doi: 10.3389/fphar.2024.1410810 (PMC11263108; doi:10.3389/fphar.2024.1410810)
Supplement: Supplementary file 2 [file DataSheet1.docx]

***Supplemental Figures***


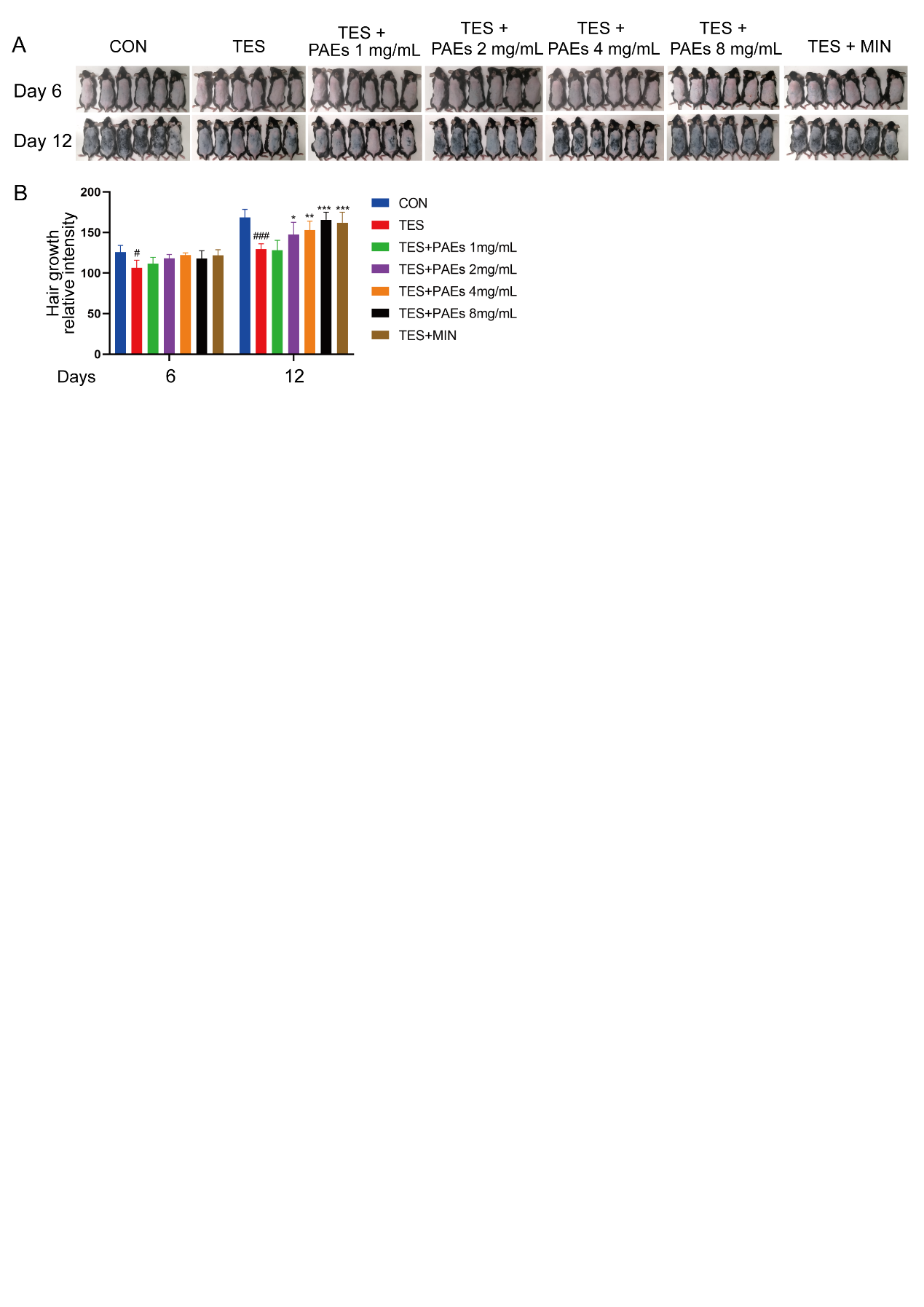


**Supplementary Figure 1.** PAEs increased hair generation in a dose-dependent way in AGA mice. (**A**) Photographs of mice in the groups of CON, TES, TES+PAEs 1, 2, 4, 8 mg/mL, and the TES+MIN on days 6 and 12. (**B**) Dorsal hair quantification on days 6 and 12 in each group. (n = 6). Comparing the CON group, ^#^*P* < 0.05, ^###^*P* < 0.001, and the TES group, ^*^*P* < 0.05, ^**^*P* < 0.01, ^***^*P* < 0.001.


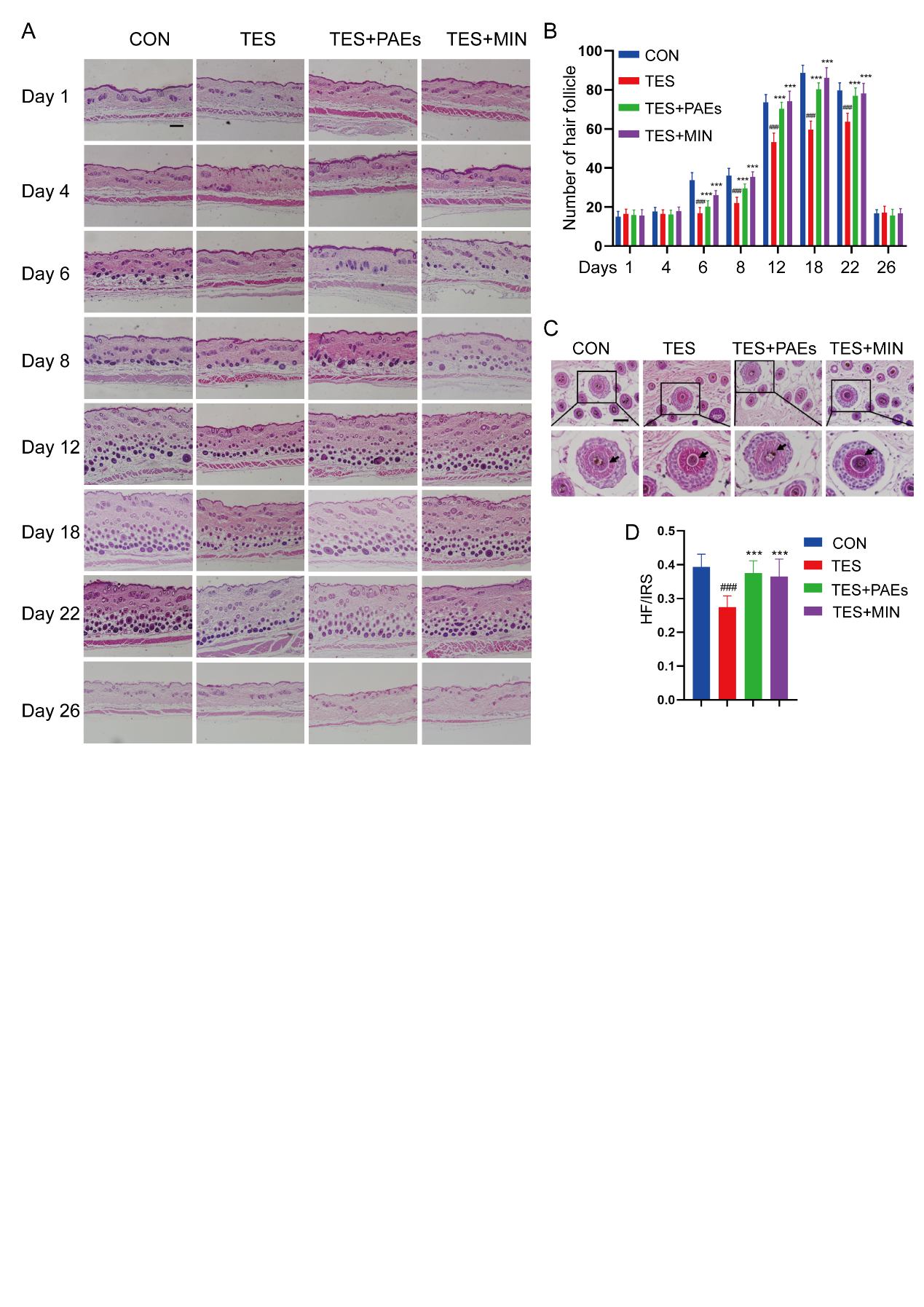


**Supplementary Figure 2.** PAEs increased HF counts and alleviated the IRS variation in AGA mice. (**A**) Representative transverse sections of mouse skin of each group stained with H&E on days 1, 4, 6, 8, 12, 18, 22, and 26. Scale bar: 200 µm. n = 3. (**B**) The number of HF were counted on days 1, 4, 6, 8, 12, 18, 22, and 26 by Image J. (**C**) Transverse slices of mice's dorsal skin stained with H&E revealed a hair shaft wrapped in IRS (indicated with black arrows) on day 12. Scale bar: 100 µm. (**D**) Quantification of guard hair's hair shaft/IRS cross-sectional diameters in **(C)** (n = 15). Comparing the CON group, ^##^*P* < 0.01, ^###^*P* < 0.001, and the TES group, ^*^*P* < 0.05, ^**^*P* < 0.01, ^***^*P* < 0.001.


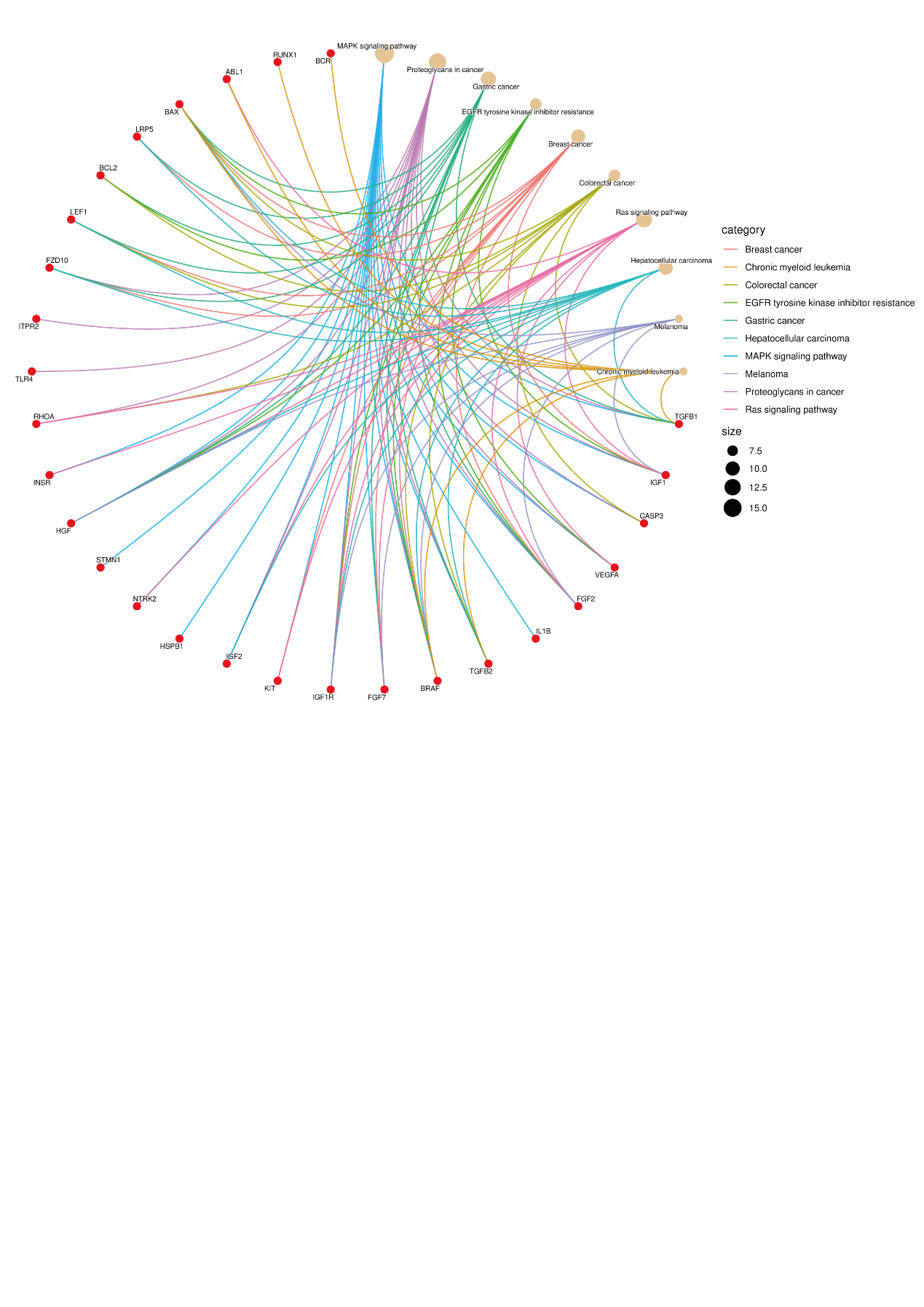


**Supplementary Figure 3.** KEGG pathway analysis of intersecting targets between PAEs active ingredient targets and AGA disease targets.


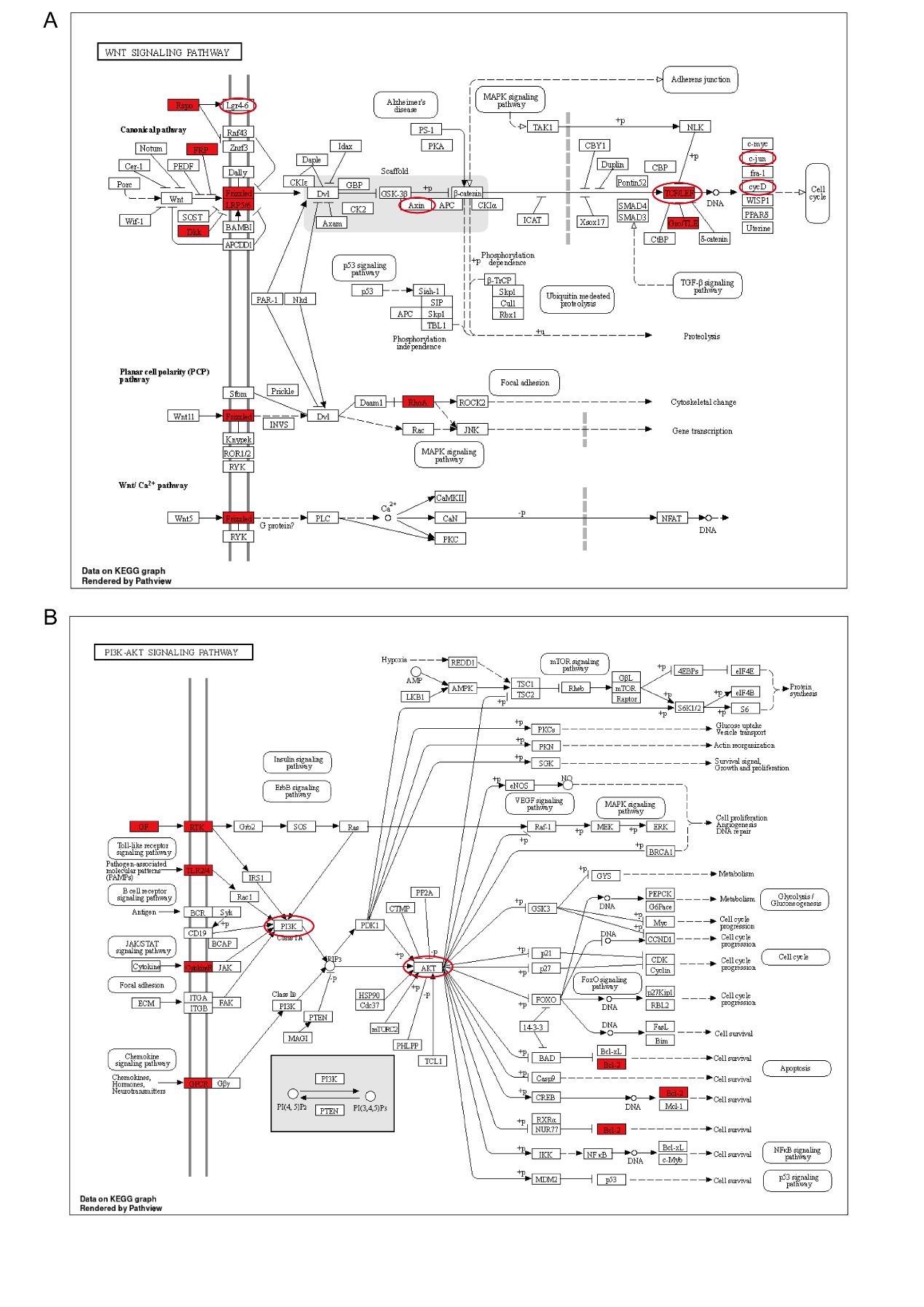


**Supplementary Figure 4.** The original KEGG pathway maps for Wnt-Catenin pathway (A) and the PI3K-AKT pathway (B). Note: The genes highlighted with red circles in (A) and (B) are the ones we tested in this study.


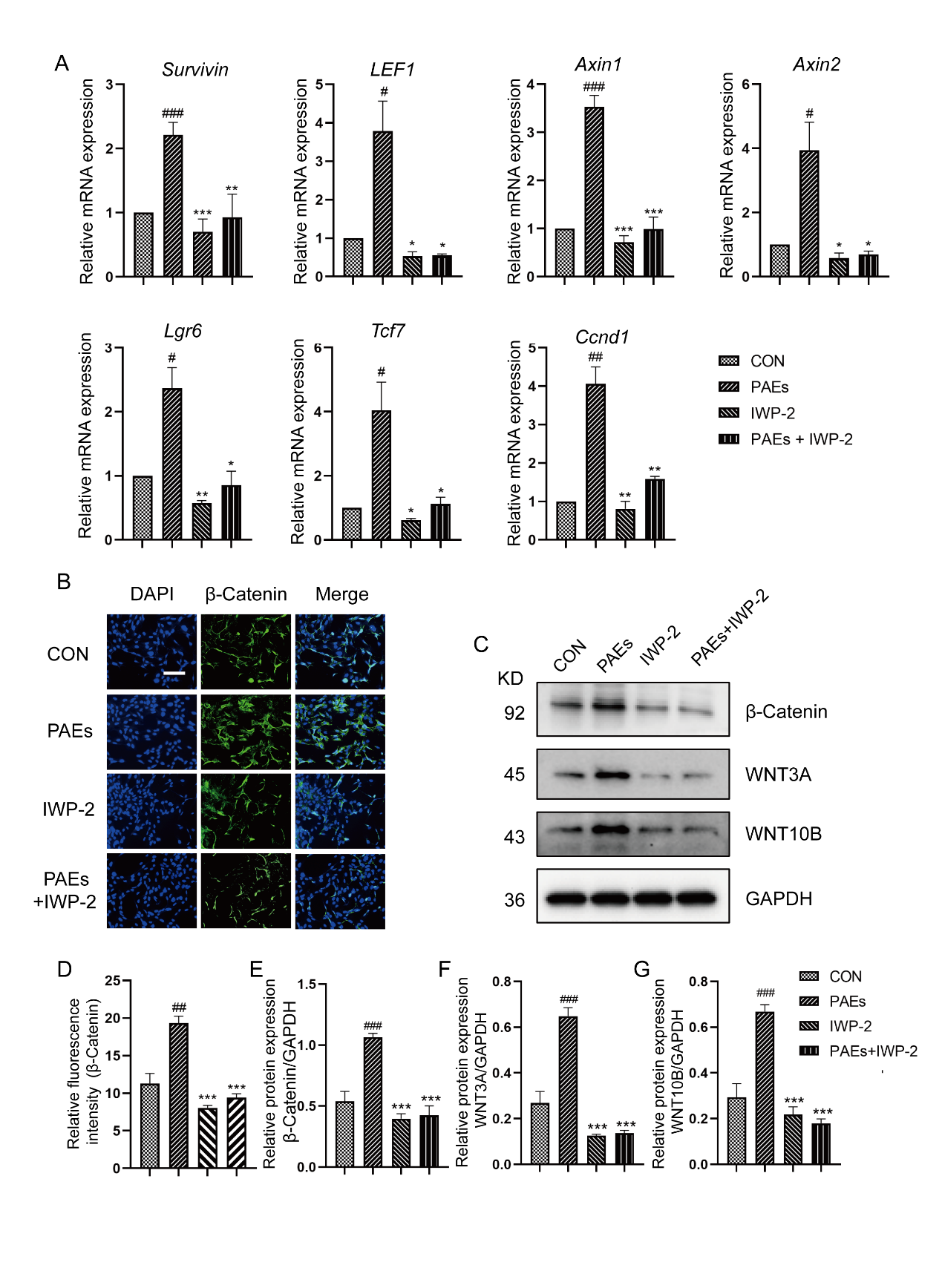


**Supplementary Figure 5.** PAEs stimulated the proliferation of HFSC by activating the Wnt-β-Catenin pathway. (A) The qRT-PCR analysis of *Survivin*, *LEF1*, *Axin1*, *Axin2*, *Lgr6*, *Tcf7*, and *Ccnd1* genes in each group after WNT pathway blockade in HFSC. (B) Representative images of immunofluorescence of β-Catenin in HFSC of each group, Scale bar: 200 μm, (C) Representative Western blot images of WNT3A, WNT10B, and β-Catenin of each group in HFSC. (D-G) Statistical analysis of immunofluorescence and Western blot images. Comparing the CON group, ^###^*P* < 0.001, and the PAEs group, ^*^*P* < 0.05, ^**^*P* < 0.01, ^***^*P* < 0.001.
